# Impaired Anti-Tumor T cell Response in Hepatocellular Carcinoma

**DOI:** 10.3390/cancers12030627

**Published:** 2020-03-08

**Authors:** Nada Chaoul, Serena Mancarella, Luigi Lupo, Gianluigi Giannelli, Francesco Dituri

**Affiliations:** 1 Department of Biomedical Sciences and Human Oncology, University of Bari Medical School, 70124 Bari, Italy; nadach@gmail.com; 2 National Institute of Gastroenterology “S. De Bellis,” Research Hospital, 70013 Castellana Grotte, Italy; serena.mancarella@irccsdebellis.it (S.M.); gianluigi.giannelli@irccsdebellis.it (G.G.); 3 General Surgery and Liver Transplantation Unit, University of Bari Medical School, 70124 Bari, Italy; luigigiovanni.lupo@uniba.it

**Keywords:** HCC, antitumor immunity, T cell response, immunotherapy

## Abstract

Different subsets of lymphocytes have the capacity to promote or counteract the progression of solid cancers, including hepatocellular carcinoma (HCC). Therefore, to determine the infiltrative ability and functional status of major immune cell subtypes into tumor may lead to novel insights from the perspective of immunotherapy. After obtaining single cell suspensions from freshly collected specimens of HCC tumor, along with paired peritumor tissues and peripheral blood mononuclear cells (PBMCs) from 14 patients, we flow-cytometrically identified and quantified the relative frequencies of lymphocyte subsets within the tissues of origin. We found that the recruitment in the tumor of cytotoxic cells, namely the terminally differentiated CD4+ and CD8+ T cells (TEFF), is impaired, whereas the effector memory CD4+ T cells (TEM) are more attracted in this site. Concerning the other subsets, the frequency of NK CD56hi and NKT CD56hi cells infiltration in the tumor is increased, whereas that of NKT CD56low is reduced. Although CD4+ and CD8+ T cells settled in the tumor show a higher degree of activation than the circulating counterpart, they occur with a more exhausted phenotype. Overall, these data demonstrate the prevalently immunosuppressive nature of HCC microenvironment, and prompt us to search for strategies to enhance the activity of anti-tumor immune cell subsets.

## 1. Introduction

Hepatocellular carcinoma (HCC) ranks as the third leading cause of cancer-related deaths worldwide and one of the most common cancers; it occurs primarily in patients with underlying chronic liver disease and cirrhosis. Until recently, HCC patients had limited treatment options, including only tumor resection, liver transplantation, thermo-ablation and trans-arterial chemoembolization (TACE), but the five-year survival is poor compared with other malignancies and the rate of tumor recurrence after surgery is very high (70–80% of cases) [1,2]. Sorafenib, a multikinase inhibitor, the only multi-targeted pharmaceutical agent, was approved for the treatment of patients with advanced HCC in 2007 [3], but it increases patient survival by only three months in advanced stage disease [4]. It also has various side effects including alopecia, arthralgia, diarrhea, fatigue, headache and hypertension [5,6]; improved therapeutic strategies are thus urgently needed. The other major problem in the treatment of HCC is the onset of resistance to chemotherapeutic drugs, and even multi-drug resistance [7,8].

The role of immunity in cancer has been established in the recent decades; many studies described the importance of the immune cell infiltrate in the tumor microenvironment of various cancer types, but also, and very importantly, the nature of infiltrating cells and their interactions with the tumor [9,10], which itself evades immune control and creates an immunosuppressive microenvironment. Indeed, the intra-tumoral accumulation of immunosuppressive cells, such as myeloid-derived suppressor cells or regulatory T cells (Tregs), has been well documented in patients and in animal models and is considered of bad prognosis in most cancers [11,12,13,14,15,16]. Furthermore, increased frequencies of circulating Tregs were observed in HCC patients and were indeed associated with low survival [17]. A recent study also showed a significant increase in the frequency of Foxp3+ CD8+ T cells, that correlated with the elevated frequencies of peripheral blood Tregs and with the stage of the disease in HCC patients [18]. On the contrary, the infiltration of the tumor by T helper 1 (Th1) cells and cytotoxic CD8+ T lymphocytes (CTL) is usually of good prognosis [9,10]. However, these tumor-infiltrating CTL are usually exhausted: they express negative regulators of T cell activity also known as “immune checkpoints”, such as PD-1 or CTLA-4 and have an impaired cytotoxic activity [19]. These observations urged pre-clinical and clinical studies to evaluate the strategies to overcome the immunosuppression and exhaustion at tumor site and enhance the anti-tumor response of cytotoxic cells. Many immunotherapeutic approaches are under evaluation for many cancers, whether used as monotherapy, combination or adjuvant therapy to approved treatments: immunotherapeutic vaccines, adoptive cell therapy [20,21,22], cytokine treatments (including in HCC patients), or immune checkpoint inhibitors [23,24], even in HCC patients [25,26].

In the particular case of HCC, immune cells are not only involved in the anti-tumoral response, but also in the pathogenesis of the tumor [27,28], since most HCC cases develop after chronic liver inflammation in response to the hepatitis viruses B or C (HBV and HCV respectively) or to cirrhosis. It has thus become urgent to clearly understand the role of each immune cell type in the initiation and progression of liver cancer, to better develop more targeted and more efficient immunotherapies.

In this study, we aimed at fully characterizing the lymphoid immune infiltrate at the tumor site as well as in the peritumoral tissue and the peripheral blood of HCC patients at different stages of the disease.

## 2. Results

### 2.1. Lymphocyte Recruitment into HCC Tumor and Peritumor Site

**Figure 1:**
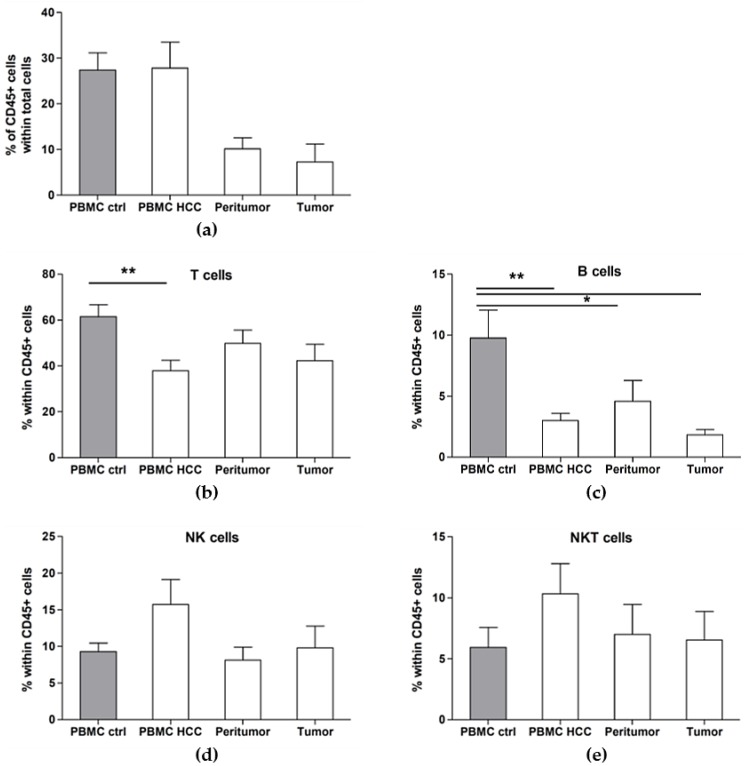
Hepatocellular carcinoma (HCC) patients have imbalanced circulating lymphocyte proportions and an impaired immune infiltration to the tissues. Cell suspensions were prepared from peripheral blood mononuclear cells (PBMC), tumor and peritumor taken during surgery and were analyzed by flow cytometry. The PBMC from healthy donors were used as control. (**a**) Percentage of immune infiltration was identified by the total CD45+ cell portion in each tissue. (**b**–**e**) Percentage of lymphoid subsets within total CD45+ cells. Grey histograms represent cells obtained from healthy donors, while white histograms represent cells from HCC patients. Results are expressed as Mean ± SEM from cumulative results (n = 9–14 patients or controls). * *p* < 0.05 and ** *p* < 0.01, as determined by Mann–Whitney’s test between each lymphoid subset, in each tissue.

First, we analyzed the immune cells’ frequencies in the peripheral blood of HCC patients as compared to healthy controls. As expected, we found similar frequencies of total CD45^+^ cells in both groups (Figure 1a). However, the percentages of T cells and B cells were significantly reduced in HCC patients, while those of NK cells (CD3^−^ CD56^+^ cells) and NKT cells (CD3^+^ CD56^+^ cells) were increased (Figure 1b–e and Table 1). When we analyzed the infiltration of immune cells (CD45^+^) in the tumor tissue and peritumor, we found that the percentage of CD45^+^ cells in the peritumor is higher than that in the tumor itself (Figure 1a), showing that although the liver is considered as an immunological organ, the infiltration of immune cells to the tumor site is impaired. When we further analyzed the lymphoid subsets, we found that the immune infiltrate in the peritumor consists mainly of T cells (49.79 ± 5.76), NK cells (8.13 ± 1.76) and NKT cells (7.00 ± 2.45), while B cells were lower (4.57 ± 1.72) (Figure 1b–e and Table 1). T cell and B cell percentages were lower in the tumor site as compared to the peritumor, while NK and NKT cell frequencies were similar. These results confirm the impaired recruitment of lymphocytes to the site of the tumor: indeed, while T cells are increased in the peritumor, their entry to the tumor site seems to be inhibited. Furthermore, while the percentages of NK and NKT cells are increased in the blood of HCC patients, these cells are less present in the peritumor and not more recruited to the tumor site. These data clearly show, and corroborate previously published data [29], that the tumor site is a very particular microenvironment with preferential recruitment of some immune subsets.

### 2.2. CD56hi NK and NKT Cells Are Increased in the Tumor

**Figure 2:**
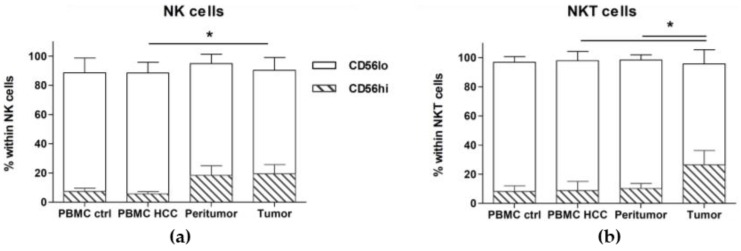
Decreased frequencies of CD56low NK and NKT cells in tumors. Cell suspensions were prepared from PBMC, tumor and peritumor taken during surgery and were analyzed by flow cytometry. The PBMC from healthy donors were used as control. The intensity of CD56 expression in NK and NKT cells was analyzed in all tissues and the expression of CD56hi (hatched histograms) and CD56lo (white histograms) NK and NKT cells are shown in (**a**) and (**b**), respectively. Results are expressed as Mean ± SEM from cumulative results (n = 9–14 patients or controls). * *p* < 0.05, as determined by Mann–Whitney’s test between each lymphoid subset, in each tissue.

When we analyzed the frequencies of NK (CD56+ CD3-) and NKT cells (CD56+ CD3+), we observed increased proportions of the CD56hi subset of NK cells, both in the peritumoral (18.49 ± 6.51) and tumoral tissues (19.6 ± 6.13), as compared to those in the peripheral blood mononuclear cells (PBMC) of the same HCC patients (5.71 ± 1.59) and in the PBMC of healthy donors (7.47 ± 2.21) (Figure 2a and Table 2). On the other hand, the proportions of CD56low NK cells were decreased in both tissues. However, the proportion of CD56hi NKT cells was only increased in the tumor (Figure 2b and Table 2), while the CD56low NKT cells were decreased. Since the CD56low subset of NK cells is known for their cytotoxic activity [30], we can suggest that in the tumor microenvironment, the cytotoxic NK cells are reduced.

### 2.3. Cytotoxic T Cells Are Reduced at Tumor Site, While Tregs Accumulate

**Figure 3:**
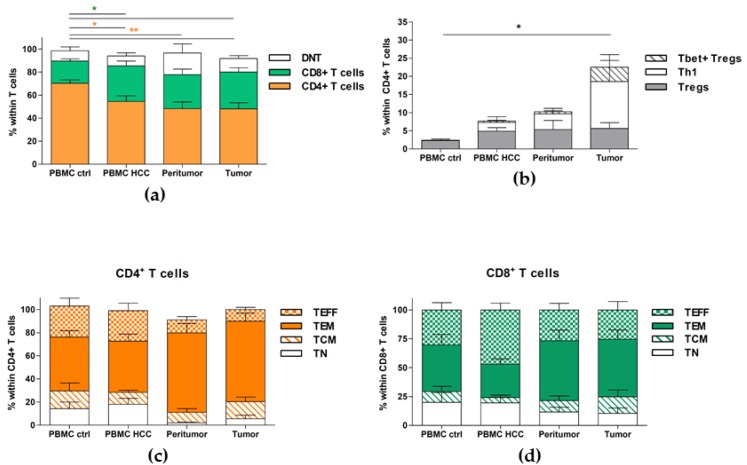
Tregs are increased in HCC patients while Effector T cells are reduced. Cell suspensions were prepared from PBMC, tumor and peritumor taken during surgery and were analyzed by flow cytometry. The PBMC from healthy donors were used as control. The frequencies of T cell subsets were analyzed in detail. (**a**) Frequencies of total CD4^+^ T cells, and CD8^+^ T cells within total CD3^+^ T cells are shown; (**b**) Frequencies of Tregs (CD4^+^ Foxp3^+^ T-bet^−^ T cells), Th1 (CD4^+^ Foxp3^−^ T-bet^+^ cells) and T-bet^+^ Tregs (CD4^+^ Foxp3^+^ T-bet^+^ cells) within CD4^+^ T cells. (**c**,**d**) Naïve (T_N_), central memory (T_CM_), effector memory (T_EM_) and effector cell (T_EFF_) subsets within CD4^+^ T cells and CD8^+^ T cells. Results are expressed as Mean ± SEM from cumulative results (with n = 9–14 patients or controls). * *p* < 0.05 and ** *p* < 0.01 as determined by Mann–Whitney’s test between each lymphoid subset, in each tissue.

We further analyzed the T cells subsets in all tissues and observed an increased percentage of CD8+ T cells in PBMC of HCC patients, as compared to controls, while the CD4+ T cells were reduced (Figure 3a and Table 3). The frequencies of CD8+ T cells in the peritumor and in the tumor where similar to the PBMC of the same patients; however, the frequencies of CD4+ T cells were similar in both tissues, but lower than in their blood. Due to the fact that regulatory T cells (Tregs) are known to be increased in cancer patients [31,32,33], we also evaluated Tregs (CD3+ CD4+ Foxp3+) in all the tissues of HCC patients and, indeed, observed an increased frequency in the PBMC of HCC patients (4.88 ± 0.97), as compared to healthy controls (2.28 ± 0.44) (Figure 3b and Table 3). Their proportion was even higher in the peritumor and in the tumor (5.32 ± 2.50 and 5.63 ± 1.59, respectively). These data show that Tregs have an increased recruitment to tumor site and are not blocked in the peritumor tissue. Th1 cells (CD3+ CD4+ T-bet+ Foxp3-) are also increased in the PBMC of HCC patients (2.41 ± 1.60), and even more increased in their peritumoral tissue (4.35 ± 1.49) and at the tumor site (12.97 ± 7.39) (Figure 3b and Table 3); showing that the tumor microenvironment is an ongoing inflammatory response. Furthermore, we detected the presence of T-bet+ Tregs (CD3+ CD4+ T-bet+ Foxp3+) only at tumor site.

### 2.4. Terminally Differentiated Effector T Cells Are Strongly Reduced at Tumor Site

We wanted to further analyze the compartmentalization of naive, effector and memory cells within CD4+ and CD8+ T cells. We used the CD45RA and the CCR7 markers to distinguish between naïve T cells (TN; CD45RA+ CCR7+), central memory T cells (TCM; CD45RA- CCR7+), effector memory T cells (TEM; CD45RA- CCR7-) and terminally differentiated effector T cells (TEFF; CD45RA+ CCR7-) (representative dot plots are shown in Supplementary Figure S1, left panels). We found that the proportions of CD4+ TN were slightly increased in the blood of HCC patients as compared to healthy controls (18.06 ± 5.21 vs. 14.24 ± 5.80), and TCM were decreased (10.24 ± 1.84 vs. 15.36 ± 6.87 in controls) (Table 4 and Figure 3c), whereas the proportions of TEM and effector cells TEFF were similar. On the contrary, the percentages of TN were significantly decreased in the peritumor and at the tumor site (2.02 ± 0.66 and 5.55 ± 3.22, respectively). TCM are similar in the peritumor and the PBMC of the same patients but were surprisingly higher in the tumor (8.99 ± 3.27 and 14.69 ± 4.14, respectively). TEM percentages were significantly increased in both the peritumor and tumor (68.8 ± 8.11 and 69.75 ± 6.98, respectively), while the terminally differentiated TEFF were decreased in both tissues (11.22 ± 2.8 and 9.95 ± 1.95, respectively), as compared to PBMC of the same patients (26.22 ± 6.52) and to the PBMC of healthy donors (26.82 ± 6.79). When we look at these subsets within CD8+ T cells, we observe that while TN in the PBMC of HCC patients (19.47 ± 4.99) were similar to those of healthy donors (19.90 ± 8.58) (Table 4 and Figure 3d), and both TCM (4.62 ± 2.10) and TEM (28.98 ± 4.64) were decreased as compared to controls (9.36 ± 4.39 and 40.31 ± 9.17, respectively). Importantly, TEFF were drastically increased in the PBMC of HCC patients (46.93 ± 5.90 vs. 30.43 ± 6.32, in healthy controls). In the peritumor, we found decreased proportions of TN (11.56 ± 4.2), but TCM were higher than in patients’ PBMC (9.86 ± 3.99). TEM were significantly increased (51.91 ± 9.23) as compared to the PBMC, while TEFF were decreased (26.68 ± 5.78). In the tumor, however, TN were lower than in patients’ PBMC but similar to the peritumor (10.45 ± 4.61), while TCM were strongly increased (14.3 ± 5.88). TEM were augmented similarly to the peritumor (49.77 ± 8.16). Most interestingly, TEFF were lower than in the PBMC, but similar to the peritumor (25.49 ± 7.26). These data corroborate our hypothesis that some subsets are preferentially recruited to the tumor site and show that the recruitment of terminally differentiated effector T cells to the tissue is strongly impaired.

### 2.5. Tumor- and Peritumor-Infiltrating T Cells Are Activated and Exhausted

**Figure 4:**
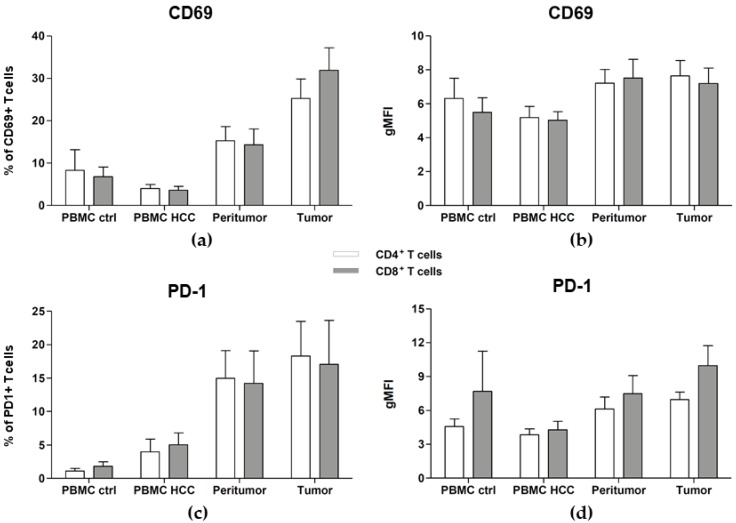
The percentage of PD-1^+^ and CD69^+^ T cells are increased in the tumor. Cell suspensions were prepared from PBMC, tumor and peritumor taken during surgery and were analyzed by flow cytometry. PBMC from healthy donors were used as control. The expression of PD-1 and CD69 on T cells from PBMC, tumor and peritumor of each patient and on T cells from PBMC of healthy controls was quantified. (**a**) Percentage of CD69^+^ CD4^+^ T cells and CD-69^+^ CD8^+^ T cells; (**b**) gMFI of CD69; (**c**) Percentage of PD-1^+^ CD4^+^ T cells and PD-1^+^ CD8^+^ T cells; (**d**) Geometric mean fluorescence intensity (gMFI) of PD-1. Results are expressed as Mean ± SEM from cumulative results (with n = 9–14 patients or controls). See Table 5 for statistical analysis.

Finally, because T cells that infiltrate tumor sites were previously shown to be exhausted [32], we checked their activation state using the CD69 and the PD-1 markers (representative dot plots are shown in supplementary Figure S1, right panels). We found significantly increased proportions of activated CD69+ CD4+ and CD8+ T cells in the peritumor and tumor (15.25 ± 3.38 and 25.26 ± 4.55 respectively for CD4+ T cells and 14.3 ± 3.74 and 31.87 ± 5.34 respectively for CD8+ T cells), as compared to the PBMC of the same patients and of healthy controls (Figure 4a and Table 5) while the expression intensity was only slightly higher as compared to the PBMC of healthy controls and PBMC of the same patients (Figure 4b and Table 5). Interestingly, the percentage of T cells that express PD-1 is significantly increased in the peritumor (14.98 ± 4.14 for CD4+ T cells and 14.24 ± 4.82 for CD8+ T cells), and even more at tumor site (18.3 ± 5.2 for CD4+ T cells and 17.07 ± 6.59 for CD8+ T cells) (Figure 4c and Table 5), and the intensity of the expression is also significantly increased on both T cells in the tumor as compared to the PBMC of the same patients and to those of healthy donors (Figure 4d and Table 5). These data clearly show that the tumor-infiltrating T cells are indeed exhausted.

## 3. Discussion

HCC is the most common liver cancer with very few treatment options; indeed, surgery, including tumor resection and liver transplantation, is proposed at early stages of the disease, while more advanced stages are treated with sorafenib or TACE. Traditional chemotherapy has a limited effect on HCC, especially in advanced phases. Many studies are evaluating the combination of sorafenib with TACE, or with other chemotherapeutic agents already in use for the treatment of other malignancies, or with other molecules that target cell proliferation or angiogenesis [34]. Other possible strategies, currently under evaluation for HCC, include immunotherapy using mainly checkpoint inhibitors that interfere with CTLA-4 or PD-1 pathways [34]. These inhibitors indeed showed good efficacy in other malignancies, including melanoma and renal cell carcinoma [35,36], and many were approved by the FDA [37]. However, one of the main risks in the use of checkpoint inhibitors would be the induction of autoimmune side effects known as immune related adverse events [37,38,39], particularly in the liver.

Indeed, the liver plays a major role in the clearance of gut-derived and blood-borne microbes and pathogens, through the complex network of immune cells (mainly NK cells and lymphocytes) and non-parenchymal liver-resident cells (Kupffer cells, dendritic cells, liver sinusoidal endothelial cells), that create an immunosuppressive microenvironment via the production of anti-inflammatory cytokines including Interleukin-10 and TGF-β. The liver is thus responsible for systemic immunotolerance to non-pathogenic microbes/antigens and to chronic inflammation and the deregulation of this network leads to loss of tolerance, liver disease (chronic infection, cirrhosis and eventually HCC) and failure. The mechanisms responsible for the altered liver function are not completely understood. Furthermore, in the case of HCC, immune cells are not only responsible for the anti-tumoral response but themselves contribute to carcinogenesis, particularly CD8+ T-cells. During the infection with HBV or HCV, virus-specific CD8+ T-cells are responsible for the clearance of the virus, however, these cells become somehow exhausted and immune suppressed by the liver environment and thus are unable to eliminate the pathogens, resulting in chronicity and HCC. In addition, myeloid-derived suppressor cells (MDSCs) also represent an important immunosuppressive branch of the immune infiltrate in HCC. They are a heterogeneous pool of leukocytes that are massively recruited to the tumor site, due to the hypoxia-induced activation of surface enzymes and release of chemokines [40,41]. From a therapeutic perspective, more efficient interventional framework may arise from combining current immune treatments based on PD1, PD-L1, and CTLA-4 inhibition, with the induction of MDSCs differentiation or activity neutralization [42]. Thus, manipulating the immune system or certain immune cells as a therapy for HCC should be studied carefully, and understanding the immune response is crucial.

Therefore, in our study, we aimed at fully characterizing the lymphoid immune response to HCC at different stages, in the blood, the tumor and the peritumor.

We found significant changes in the distribution of immune cells in the peripheral blood of HCC patients as compared to healthy controls, but more importantly, some immune subsets were preferentially recruited to the peritumor or to the tumor itself, while other cells were not. Indeed, total T-cells and B cells were decreased in the PBMC of HCC patients as compared to healthy donors, but not in the tissues; similarly, NK and NKT cells were only increased in the blood of HCC patients, but not in either tissues.

Total CD4+ T cells were decreased in HCC patients, in all organs, while the CD8+ T cells were increased. These results seem comforting as the infiltration of CTL is usually of good prognosis for the patients [9,10]. When we looked further into the T cells subsets, we found reduced TEM in the PBMC of HCC patients as compared to healthy controls, while the terminally differentiated effectors (TEFF) were significantly decreased in the peritumor and tumor as compared to the PBMC of the same patients, confirming the preferential recruitment of some immune subsets to the tumor. These data also clearly show, that although the immune response seems to be mounted in the periphery, some mechanism(s) seem(s) to inhibit the migration or the entry of cytotoxic and effector cells to the tumor site, partly explaining why the anti-tumoral immune response is impaired in the tissue. Indeed, the cytotoxic cells (including NK, NKT and CD8+ TEFF) are activated and expanding in the periphery, but seem unable to reach the site of inflammation. Therefore, understanding the mechanisms responsible for the altered recruitment to tumor site is crucial. The significantly decreased proportions of the cytotoxic CD56lo subset [30] of NK and NKT cells in the peritumor and furthermore in the tumor, corroborate the reduced efficacy of the cytotoxic response to the tumor. 

We also found increased frequencies of Th1+ CD4+ T cells in HCC patients, particularly in the tumor itself, showing an efficient recruitment of these cells and an ongoing cytotoxic response in the tumor. However, Tregs were also highly increased in the PBMC of HCC patients and in both tissues, with no differential recruitment. From the light of many studies, the accumulation of Tregs is usually of bad prognosis [11,12,13,14,15,16]. Moreover, we observed a preferential accumulation of T-bet+ Tregs in the tumor tissue, while they are rare in PBMC and peritumor of HCC patients, and absent in healthy controls. Recent studies described these Tregs, and showed that they are able to dynamically express the transcription factor of the cells they are supposed to inhibit [43,44]. In our case, we can also suggest that a part of the Tregs accumulating in the tumor are aimed at inhibiting Th1+ CD4+ T cells. Additionally, the preferential accumulation of total Tregs (both T-bet+ Foxp3+ and T-bet- Foxp3+ Tregs) might be due to the presence of a gradient of cytokines that attract them, including TGF-β. TGF-β signalingplays indeed a major role in liver disease onset and progression [45], but also in the differentiation and function of Tregs. It cannot be excluded that once Th1 cells reach tumor site, they convert into Tregs, due to the immunosuppressive microenvironment. Furthermore, Tregs exert immune suppression through the production of anti-inflammatory cytokines including TGF-β and IL-10 or through the expression of surface molecules, such as CTLA-4. Therefore, TGF-β is playing a crucial role in HCC development and progression. Studies are currently evaluating the way to target TGF-β as a therapy for HCC [46]; Galunisertib, an inhibitor of TGF-β, is currently in a phase II clinical trial for HCC patients. Notably, in a recent report, a positive correlation between CD4^+^, CD8^+^ FoxP3^+^ Treg cells and the progression stage of HCC was also observed. This association meaningfully enforces the significance of evaluating the whole Treg compartment for a more reliable assessment of disease staging [18]. Furthermore, we found increased proportions of T cells that express the activation marker CD69 in the PBMC of HCC patients; they were more significantly increased in the tissues, clearly showing the activated status of T cells. On the other hand, significantly increased percentages of PD-1+ T cells were also found in both tissues, but not in the PBMC of HCC patients, confirming that these cells are activated but, once in the tissue, they become unable to eliminate tumor cells, probably due to the chronic inflammation or to the immunosuppressive liver microenvironment. Our results are in line with those obtained by Zheng et al., who performed a single-cell characterization of the transcriptome of T cells, in the blood, tumor and surrounding tissue in 6 HCC patients infected with the hepatitis B virus, and identified new biomarkers in tumor Tregs and exhausted CD8+ T cells [47]. It is interesting to note that in two different liver diseases (HBV- and HCV-induced HCC), in patients who develop chronic infection and progress to HCC, we observe eventually similar impairments in the adaptive immune response: an accumulation of Tregs and exhaustion of CD8+ T cells. 

These results led us to try to assess the efficacy of the cytotoxic response to the tumor in these patients. Our preliminary data show that PBMC, tumor- and peritumor-infiltrating lymphocytes are unable to kill tumor cells in vitro, but that the addition of an anti-PD-L1, but not anti-PD-1, inhibitor partly restores their cytotoxic ability (data not shown). It would therefore be very interesting to assess the combination of approved drugs such as Sorafenib with a checkpoint inhibitor.

In summary, our data suggest that the anti-tumoral immune response is likely developing normally, at least in the periphery, but is impaired or inhibited by many mechanisms that occur in the tissue. It is well known that the immune response that is mounted in the periphery is reshaped in the tissue [48], particularly the tumor tissue. Since solid tumors, particularly carcinomas, are usually less vascularized than normal tissue, the recruitment of immune cells would be reduced as compared to the peritumor or normal tissue. Moreover, less nutrients, including oxygen and glucose, are available in the tumor microenvironment. Recent studies have shown that under low-glucose conditions, Tregs, for example, can adapt and overcome the suppression of T cell metabolism and function, while conventional T cells cannot [49,50]. Therefore, we propose a model where: (i) the anti-tumoral immune response seems to mount properly in the periphery and probably in the liver tissue itself, where the inflammation is taking place, (ii) but that some immune subsets are preferentially recruited to both the peritumor and tumor site (including Th1 cells, Tregs, TEM, CD56hi NK and NKT cells), (iii) while some cells are only accumulating in the tumor itself (T-bet+ Tregs), and other cells’ entry or function is inhibited (TEFF). Once in the tissue, the microenvironment reshapes their phenotype and function and favors immunosuppression, explaining why the anti-tumoral response is impaired. 

To our knowledge, this is the first study that analyzes the changes in lymphoid response simultaneously in three different organs from each HCC patient, giving a clear overview of the peripheral and local anti-tumoral lymphoid response in HCC patients infected with the hepatitis C virus. Our next step would be to evaluate the efficacy of the anti-tumoral response and try to find the optimal combination therapy to enhance the cytotoxic response to tumor cells.

## 4. Materials and Methods

### 4.1. Patients

Peripheral blood, tumor and peritumoral tissue from 14 patients with HCC (all males aged between 52 and 74 years; suffering from HCV infection) undergoing tumor resection or liver transplant were collected at the surgery room of the Department of Emergencies and Organ Transplant of the Policlinic hospital of Bari, between September 2016 and September 2017. Tissues were taken directly after surgical removal from 13 patients (2 patients underwent thermo-ablation of the tumor, without surgical intervention and tissue collection). The peripheral blood of 9 healthy individuals was taken as control. This study has been approved by the local ethical committee of University Hospital Policlinico Consorziale and has also been accepted by the Italian Association for Cancer Research.

### 4.2. Isolation of Patients’ Peripheral Blood Mononuclear Cells (PBMC), Tumor-Infiltrating Lymphocytes (TILs) and Lymphocytes from the Peritumor Site

Blood samples were collected during surgery and processed for PBMC isolation using the Ficoll-paque Plus gradient centrifugation (GE Healthcare, Chicago, IL, USA). Briefly, after the removal of plasma, the blood was diluted in PBS 1× (1:1 volume) and layered over the ficoll-hypaque (ratio 2:1) and centrifuged for 30min at 800 rcf, without brake, at room temperature. The upper layer was then discarded and the PBMC at the interface between the PBS and the ficoll were harvested, washed, counted and further processed for FACS staining. Tumor and peritumor tissue samples were harvested, mechanically disrupted and treated for 2 to 4h with 400 U/mL collagenase D, then filtered to obtain single-cell suspensions. Lymphoid cells were further isolated using the Ficoll-paque Plus gradient centrifugation, as described for PBMC.

### 4.3. Flow Cytometry Analysis

The following monoclonal antibodies (mAbs) were used for FACS staining: FITC-conjugated anti-FoxP3, or anti-CCR7, APC- conjugated anti-CD56, or anti-CD69, or anti-CD127, APC-eF780-conjugated anti-CD3, AlexaFluor 700-conjugated anti-CD4, eFluor450-conjugated anti-CD8, PerCPCy5.5-conjugated anti-CD45RA or anti-HLA-A2, PE-conjugated anti-CD19 or anti-PD-1, PE-Cy7-conjugated anti-CD45. PBMC, tumor and peritumor cell suspensions were incubated for 20min with the antibody mixes in the dark at 4 °C, washed twice. For the staining and detection of Th1 and Tregs, cells were permeabilized and stained with PerCPCy5.5- conjugated anti-T-bet and FITC-conjugated anti-Foxp3, respectively, according to the manufacturer’s protocol (eBioscience Thermo Fisher Scientific, Waltham, MA, USA). All mAbs and their respective isotypes were purchased from BD Pharmingen and eBioscience. Stained cells were then acquired on a Navios cytometer (Beckman coulter, Brea, CA, USA) and analyzed using the Flowjo^TM^ software (Becton Dickinson and Company, Ashland, OR, USA) (Supplementary Figure S2).

### 4.4. Statistical Analysis

The two-tailed Mann–Whitney’s test was used to measure the statistical significance for differences between the groups, using the Prism software (GraphPad Software, Inc., San Diego, CA, USA). *p* values less than 0.05 were considered statistically significant.

## 5. Conclusions

The data here presented highlight the immunosuppressive nature of HCC micro-environment. We show that the overall number of circulating T cells is reduced in HCC patients, as compared to healthy subjects. Moreover, we show that the entry of cytotoxic lymphocytes, such as CD4+ and CD8+ TEFF into the tumor tissue is compromised, and that, following recruitment, these effectors undergo exhaustion. This may be, at least in part, explained by the aptitude of solid neoplasms such as HCC to re-shape the phenotypes of lymphocytes functionally committed to exert anti-tumor activities, to finally achieve the evasion of cancerous cells from immune surveillance. Further investigations will be important to clarify the cellular and molecular mechanisms behind the impaired recruitment of immune effector cells within the tumor and/or their early exhausted status. The resulting knowledge will potentially address the major issue of designing more focused therapies to re-enable the anti-tumor immunity.

## Figures and Tables

**Table 1:** Total lymphocyte and NK subsets.

	% Within CD45^+^ Cells									
	Mean ± SEM									
	PBMC Ctrl	PBMC HCC	*p* Value ^1^	Peritumor Lymphocytes	*p* Value ^1^	*p* Value ^2^	TILs HCC	*p* Value ^1^	*p* Value ^2^	*p* Value ^3^
T cells	61.52 ± 5.16	37.89 ± 4.59	**0.0071**	49.79 ± 5.76	0.22	0.11	42.29 ± 7.12	0.09	0.38	0.51
NKT cells	5.94 ± 1.64	10.32 ± 2.48	0.36	7.001 ± 2.45	0.95	0.47	6.53 ± 2.34	0.97	0.31	0.70
NK cells	9.28 ± 1.15	15.72 ± 3.41	0.60	8.133 ± 1.76	0.68	0.15	9.80 ± 2.97	0.40	0.41	0.76
B cells	9.76 ± 2.30	3.00 ± 0.58	**0.0046**	4.569 ± 1.72	**0.032**	0.67	1.81 ± 0.45	**0.002**	0.28	0.39

*p* value ^1^ = PBMC in ctrl vs. other tissues, *p* value ^2^ = PBMC in HCC patients vs. other tissues, *p* value ^3^ = Tumor vs. peritumor.

**Table 2:** NK and NKT subsets.

	**% Within NK Cells**									
	**Mean ± SEM**									
	**PBMC Ctrl**	**PBMC HCC**	***p* Value ^1^**	**Peritumor Lymphocytes**	***p* Value ^1^**	***p* Value ^2^**	**TILs HCC**	***p* Value ^1^**	***p* Value ^2^**	***p* Value ^3^**
CD56^hi^	7.47 ± 2.21	5.71 ± 1.59	0.47	18.49 ± 6.51	0.55	0.13	19.60 ± 6.13	0.07	**0.014**	0.78
CD56^low^	81.15 ± 10.16	82.73 ± 7.43	0.78	76.45 ± 6.36	0.25	0.21	70.76 ± 8.65	0.11	0.10	1.00
	**% Within NKT Cells**									
	**Mean ± SEM**									
	**PBMC Ctrl**	**PBMC HCC**	***p* Value ^1^**	**Peritumor Lymphocytes**	***p* Value ^1^**	***p* Value ^2^**	**TILs HCC**	***p* Value ^1^**	***p* Value ^2^**	***p* Value ^3^**
CD56^hi^	8.27 ± 3.87	8.83 ± 6.38	0.35	10.28 ± 3.56	0.93	0.29	26.49 ± 9.90	0.11	**0.013**	0.14
CD56^low^	88.49 ± 3.98	88.99 ± 6.42	0.43	88.15 ± 3.49	1.00	0.28	69.19 ± 9.61	0.08	**0.003**	**0.04**

*p* value ^1^ = PBMC in ctrl vs. other tissues, *p* value ^2^ = PBMC in HCC patients vs. other tissues, *p* value ^3^ = Tumor vs. peritumor.

**Table 3:** CD4^+^ and CD8^+^ T cell subsets.

	**% Within CD3^+^ T Cells**									
	**Mean ± SEM**									
	**PBMC Ctrl**	**PBMC HCC**	***p* Value ^1^**	**Peritumor Lymphocytes**	***p* Value ^1^**	***p* Value ^2^**	**TILs HCC**	***p* Value ^1^**	***p* Value ^2^**	***p* Value ^3^**
CD4^+^ T cells	70.29 ± 2.94	54.45 ± 4.97	**0.036**	48.13 ± 6.03	**0.0056**	0.74	48.07 ± 5.22	**0.006**	0.74	0.78
CD8^+^ T cells	19.20 ± 2.08	30.97 ± 4.06	**0.0498**	29.514 ± 4.94	0.25	0.81	31.95 ± 3.65	**0.017**	0.48	0.72
	**% Within CD4^+^ T Cells**									
	**Mean ± SEM**									
	**PBMC Ctrl**	**PBMC HCC**	***p* Value ^1^**	**Peritumor Lymphocytes**	***p* Value ^1^**	***p* Value ^2^**	**TILs HCC**	***p* Value ^1^**	***p* Value ^2^**	***p* Value ^3^**
Tregs	2.28 ± 0.44	4.88 ± 0.97	0.60	5.32 ± 2.50	0.833	0.596	5.63 ± 1.59	0.024	0.840	0.463
Th1	0.04 ± 0.02	2.41 ± 1.60	0.12	4.35 ± 1.49	**0.017**	0.120	12.97 ± 7.39	0.024	0.229	0.779
T-bet+ Tregs	0.09 ± 0.01	0.40 ± 0.19	0.88	0.56 ± 0.28	0.617	0.410	3.90 ± 1.90	0.2788	0.16	0.462

*p* value ^1^ = PBMC in ctrl vs. other tissues, *p* value ^2^ = PBMC in HCC patients vs. other tissues, *p* value ^3^ = Tumor vs. peritumor

**Table 4:** T cell subsets. TEFF = Terminally differentiated CD4+ and CD8+ effector T cells. TEM = Effector memory CD4+ T cells.

	**% Within CD4^+^ T Cells**									
	**Mean ± SEM**									
	**PBMC Ctrl**	**PBMC HCC**	***p* Value ^1^**	**Peritumor Lymphocytes**	***p* Value ^1^**	***p* Value ^2^**	**TILs HCC**	***p* Value ^1^**	***p* Value ^2^**	***p* Value ^3^**
TN	14.24 ± 5.80	18.06 ± 5.21	0.65	2.02 ± 0.66	**0.0096**	**0.005**	5.55 ± 3.22	**0.030**	**0.015**	0.69
TCM	15.36 ± 6.87	10.24 ± 1.84	0.65	8.99 ± 3.27	0.46	0.52	14.69 ± 4.14	0.84	0.69	0.35
TEM	46.54 ± 5.58	44.41 ± 6.02	0.92	68.80 ± 8.11	**0.012**	**0.012**	69.75 ± 6.98	**0.020**	**0.007**	0.92
TEFF	26.82 ± 6.79	26.22 ± 6.52	0.60	11.22 ± 2.80	0.10	**0.015**	9.95 ± 1.95	0.09	**0.018**	0.97
	**% Within CD8^+^ T Cells**									
	**Mean ± SEM**									
	**PBMC Ctrl**	**PBMC HCC**	***p* Value ^1^**	**Peritumor Lymphocytes**	***p* Value ^1^**	***p* Value ^2^**	**TILs HCC**	***p* Value ^1^**	***p* Value ^2^**	***p* Value ^3^**
TN	19.90 ± 8.58	19.47 ± 4.99	0.43	11.56 ± 4.20	0.90	0.15	10.45 ± 4.61	0.94	0.06	0.70
TCM	9.36 ± 4.39	4.62 ± 2.10	0.19	9.86 ± 3.99	0.50	**0.05**	14.30 ± 5.88	0.90	0.31	0.76
TEM	40.31 ± 9.17	28.98 ± 4.64	0.32	51.91 ± 9.23	0.36	**0.049**	49.77 ± 8.16	0.36	0.10	0.85
TEFF	30.43 ± 6.32	46.93 ± 5.90	0.09	26.68 ± 5.78	0.72	**0.021**	25.49 ± 7.26	0.41	**0.023**	0.70

*p* value ^1^ = PBMC in ctrl vs. other tissues, *p* value ^2^ = PBMC in HCC patients vs. other tissues, *p* value ^3^ = Tumor vs. peritumor

**Table 5:** PD-1 and CD69 expression on T cell subsets.

	**% of CD69^+^ Cells**									
	**Mean ± SEM**									
	**PBMC Ctrl**	**PBMC HCC**	***p* Value ^1^**	**Peritumor Lymphocytes**	***p* Value ^1^**	***p* Value ^2^**	**TILs HCC**	***p* Value ^1^**	***p* Value ^2^**	***p* Value ^3^**
CD4+ T cells	8.31 ± 4.83	4.01 ± 0.96	0.64	15.25 ± 3.38	**0.0057**	**0.014**	25.26 ± 4.55	**0.0039**	**0.010**	0.65
CD8+ T cells	6.79 ± 2.30	3.60 ± 0.92	0.38	14.30 ± 3.74	**0.0006**	**0.026**	31.87 ± 5.34	**0.037**	0.12	0.55
	**gMFI of CD69^+^ Cells**									
	**Mean ± SEM**									
	**PBMC Ctrl**	**PBMC HCC**	***p* Value ^1^**	**Peritumor Lymphocytes**	***p* Value ^1^**	***p* Value ^2^**	**TILs HCC**	***p* Value ^1^**	***p* Value ^2^**	***p* Value ^3^**
CD4+ T cells	6.31 ± 1.18	5.19 ± 0.65	0.55	7.20 ± 0.82	0.70	0.10	7.65 ± 0.90	0.36	0.054	0.74
CD8+ T cells	5.50 ± 0.87	5.04 ± 0.50	0.59	7.52 ± 1.10	0.16	0.05	7.19 ± 0.90	0.18	**0.099**	1.00
	**% of PD1^+^ Cells**									
	**Mean ± SEM**									
	**PBMC Ctrl**	**PBMC HCC**	***p* Value ^1^**	**Peritumor Lymphocytes**	***p* Value ^1^**	***p* Value ^2^**	**TILs HCC**	***p* Value ^1^**	***p* Value ^2^**	***p* Value ^3^**
CD4+ T cells	1.12 ± 0.39	3.97 ± 1.95	0.92	14.98 ± 4.14	0.058	**0.0045**	18.30 ± 5.20	**0.0033**	**<0.0001**	0.20
CD8+ T cells	1.83 ± 0.67	5.04 ± 1.76	0.26	14.24 ± 4.82	0.09	**0.017**	17.07 ± 6.59	**0.0004**	**0.0001**	**0.035**
	**gMFI of PD1^+^ Cells**									
	**Mean ± SEM**									
	**PBMC Ctrl**	**PBMC HCC**	***p* Value ^1^**	**Peritumor Lymphocytes**	***p* Value ^1^**	***p* Value ^2^**	**TILs HCC**	***p* Value ^1^**	***p* Value ^2^**	***p* Value ^3^**
CD4+ T cells	4.58 ± 0.65	3.85 ± 0.51	0.30	6.12 ± 1.08	0.15	0.11	6.97 ± 0.67	**0.0016**	**0.005**	0.23
CD8+ T cells	7.69 ± 3.56	4.28 ± 0.75	0.19	7.48 ± 1.63	0.21	0.05	9.97 ± 1.76	**0.010**	**0.001**	0.17

*p* value ^1^ = PBMC in ctrl vs. other tissues, *p* value ^2^ = PBMC in HCC patients vs. other tissues, *p* value ^3^ = Tumor vs. peritumor

## Supplementary Materials

The following are available online at https://www.mdpi.com/2072-6694/12/3/627/s1, Figure S1: Representative dot plots for CCR7/CD45RA and CD69/PD-1 staining in T cells, Figure S2: Lymphocyte gating strategy.
