# Supplementary material for: Impaired Anti-Tumor T cell Response in Hepatocellular Carcinoma
Source: Cancers (Basel). 2020 Mar 8;12(3):627. doi: 10.3390/cancers12030627 (PMC7139707; doi:10.3390/cancers12030627)
Supplement: Supplementary file 1 [file cancers-12-00627-s001.zip › Supplementary Figure B.pdf]

Supplementary Figure B

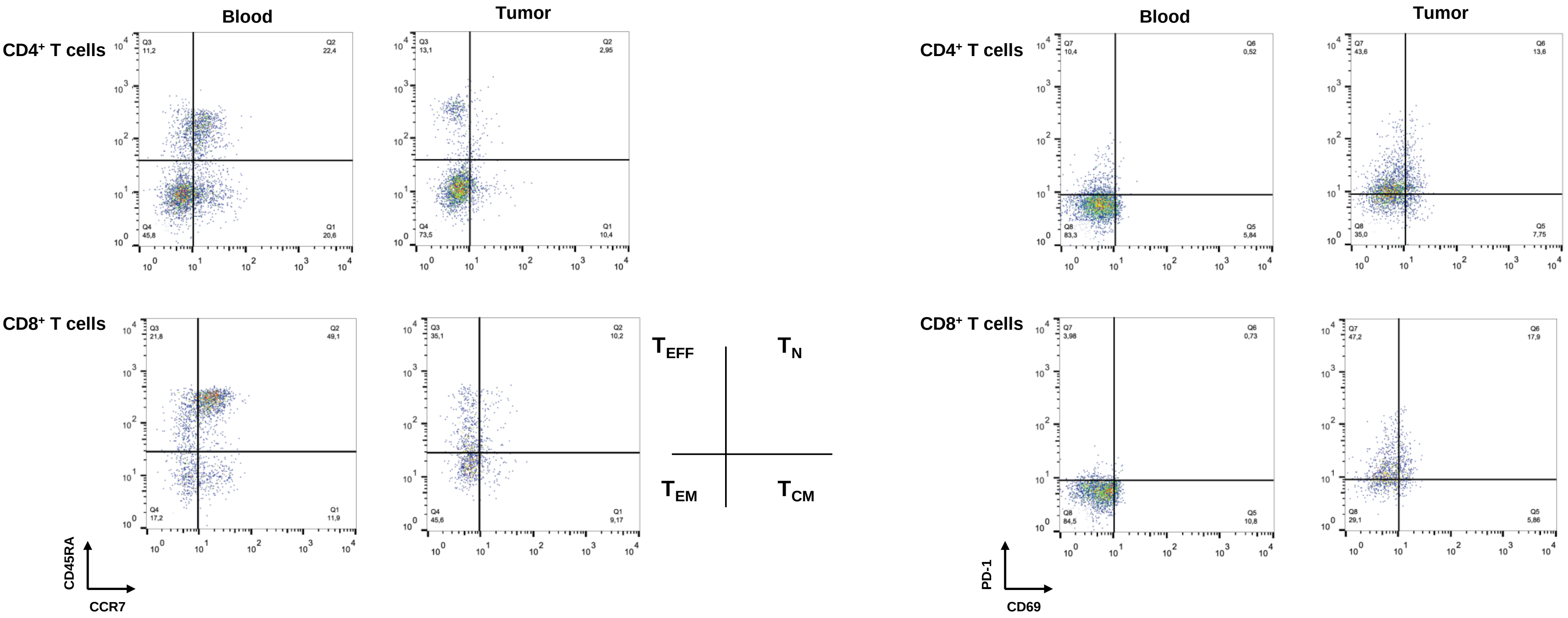

Cell suspensions were prepared from PBMC, tumor and peritumor, from HCC patients and were analyzed by flow cytometry. The PBMC from healthy donors were used as control.

Left panels: CCR7 and CD45RA staining were used to distinguish the Naïve (T_N), central memory (T_{CM}), effector memory (T_{EM}) and effector cell (T_{EFF}) subsets within $CD4^+$ T cells and $CD8^+$ T cells. One representative dot plot per subset is shown for PBMC and for tumor of one patient.

Right panels: CD69 and PD-1 staining were analyzed on CD4+ and CD8+ T cells. One representative dot plot is shown for each subset in PBMC and tumor of an HCC patient.
