# Supplementary figures and images for: Impaired Anti-Tumor T cell Response in Hepatocellular Carcinoma
Source: Cancers (Basel). 2020 Mar 8;12(3):627. doi: 10.3390/cancers12030627 (PMC7139707; doi:10.3390/cancers12030627)

Supplementary Figure A: Gating strategy

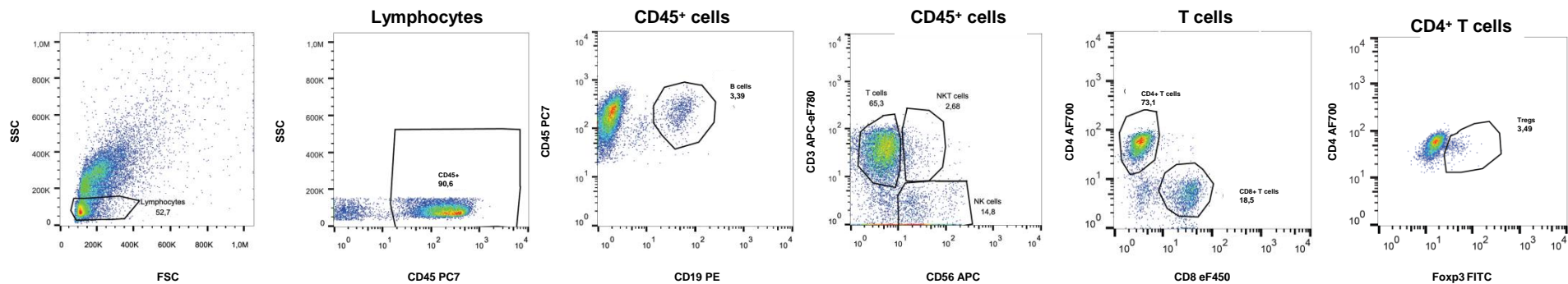

Supplement: Supplementary file 1 [file cancers-12-00627-s001.zip › Supplementary Figure A.pdf]
